# Targeting CD47/SIRPα as a therapeutic strategy, where we are and where we are headed

**DOI:** 10.1186/s40364-022-00373-5

**Published:** 2022-04-13

**Authors:** Tailong Qu, Baiyong Li, Yifei Wang

**Affiliations:** 1 College of life Science and Technology, Jinan University, No.601, West Huangpu Avenue, Guangzhou, Guangdong 510632 People’s Republic of China; 2 Department of Antibody Discovery, Akeso Biopharma, No.6 of Shennong Road, Torch Development District, Zhongshan, 528437 People’s Republic of China

**Keywords:** Immunotherapy, Phagocytosis, CD47, SIRPα, Bispecific antibody, Clinical development

## Abstract

Immunotherapy using PD-1 and CTLA4 inhibitors to stimulate T cell immunity has achieved significant clinical success. However, only a portion of patients benefit from T cell-based immunotherapy. Macrophages, the most abundant type of innate immune cells in the body, play an important role in eliminating tumor cells and infectious microbes. The phagocytic check point protein CD47 inhibits the phagocytic activity of macrophages through binding to SIRPα expressed on macrophages. Blockade of the interaction between CD47 and SIRPα could restore phagocytic activity and eliminate tumor cells in vitro and in vivo. In this manuscript, we review the mechanism of action and development status of agents (antibodies targeting CD47 and SIRPα, SIRPα-Fc fusion proteins, and bi-specific antibodies) that block CD47/SIRPα interaction in preclinical studies and in the clinical setting. In addition, small molecules, mRNA, and CAR-T/M that target the CD47/SIRPα axis are also reviewed in this article.

## Introduction

Tumor cells evade immune destruction by transmitting inhibitory signals to lymphocytes and myeloid cells [1]. Blockade of these inhibitory molecules, which include CTLA4, PD-1, and PD-ligand 1 (PD-L1), could restore T cell function and promote elimination of tumor cells. Immune checkpoint inhibitors (ICIs) have improved outcomes for patients with multiple types of cancers. However, many patients do not respond to this type of immunotherapy [2, 3]. In some cases, these therapeutic agents have been associated with disease progression [4, 5], the cause of which is currently being investigated. Therefore, drugs that act on a novel class of targets to immobilize a broader immune cell population are needed to improve upon current therapeutic options.

Macrophages [6] are typically the first dedicated innate immune cells to detect the presence of infectious pathogens or tumor cells. Macrophages are derived from monocyte precursors that circulate in the blood and migrate into tissues, after which they differentiate into tissue macrophages such as Kupffer cells in the liver, alveolar macrophages in the lung, and microglia in the brain. Circulating monocytes and resident tissue macrophages can directly kill tumor cells via phagocytosis (innate immune response) and can activate the adaptive immune response. However, these immune responses can be inhibited by ligand binding to inhibitory receptors expressed on the macrophage cell surface [7]. Signal regulatory protein alpha (SIRPα) is a transmembrane protein expressed on all myeloid cells, including monocytes, macrophages, and neutrophils. SIRPα contains immunoreceptor tyrosine-based inhibition motifs (ITIMs) that can be phosphorylated, resulting in recruitment of inhibitory molecules such as Src homology 2 (SH2) domain-containing protein tyrosine phosphatase (SHP)-1 and SHP-2 [8]. Binding of CD47 to SIRPα triggers coupling of SIRPα to these phosphatases, resulting in inhibition of phagocytic activity [9, 10]. CD47 is ubiquitously expressed on many types of cells to prevent phagocytosis by phagocytes. However, tumor cells overexpress CD47 to evade the immune system through inhibition of myeloid cell-mediated elimination [11]. Inhibition of CD47-SIRPα interaction restored the phagocytic activity of phagocytes in vitro and in vivo [12–15]. Targeting the CD47/SIRPα axis has become a promising strategy to promote tumor elimination through innate immunity. This review focuses on development, safety, and efficacy of agents that target the CD47/SIRPα axis in preclinical and clinical studies.

### CD47/SIRPα: the molecules and biology

SIRPα [16, 17], also named SHPS-1 or CD172a, is a transmembrane glycoprotein mainly expressed on neurons and myeloid cells that is particular enriched on macrophages. Human SIRPα is coded by the SHPS-1 gene located at human chromosome 20p13. The open reading frame region is composed of eight exons, including a signal peptide, extracellular domain, a transmembrane segment, and three parts of one cytoplasmic domain. The extracellular domain consists of three Ig-like regions, an NH2-terminal immunoglobulin (Ig) variable (V) region (domain 1, D1), and two Ig constant (C) regions (domain 2 and 3). The cytoplasmic region contains two immunoreceptor tyrosine based inhibitory motifs (ITIMs) and a proline-rich region (YYYY), which bind to Src homology (SH2) domain-containing molecules.

**Fig. 1:**
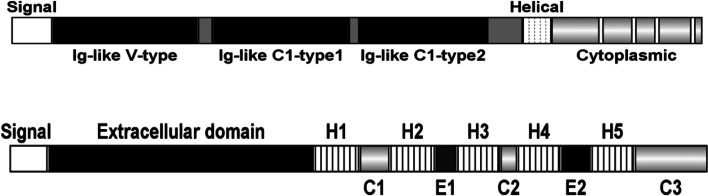
Diagram of composition of CD47 and SIRPα protein. The upper panel is the SIRPα protein with signal region (1–30), Ig-like V-type (32–137), Ig-like C1-type1 (148–247), C1-type2 (254–348), transmembrane (helical, 374–394), and cytoplasmic region (365–504) with four short spacers for SH2-binding. The lower is the CD47 protein with signal region (1–18), extracellular domain (19–141), five transmembrane region (142–289, Helical 1 to Helical 5), and cytoplasmic region (290–323)

CD47 is a 52 kD transmembrane glycoprotein belonging to the immunoglobulin superfamily. Human CD47 is encoded by the CD47 gene located at the q13.12 region of chromosome 3. Human CD47 contains an NH_2_-terminal Ig variable-like extracellular domain (ECD), a 5-transmembrane spanning helical bundle domain, and a short intracellular COOH-terminal domain (CTD) [18]. CD47 is an essential component of the innate immune system, and binding of its extracellular domain with its ligands αVβ3, SIRPα, and thrombospondin-1 (Tsp-1) activates different signaling pathways that control cell proliferation and differentiation, angiogenesis, and immune regulation. The CTD is alternatively spliced and can exist as four isoforms, ranging from 4 to 36 residues. A schematic diagram of the compositions of CD47 and SIRPα proteins are shown in Fig. 1.

**Fig. 2:**
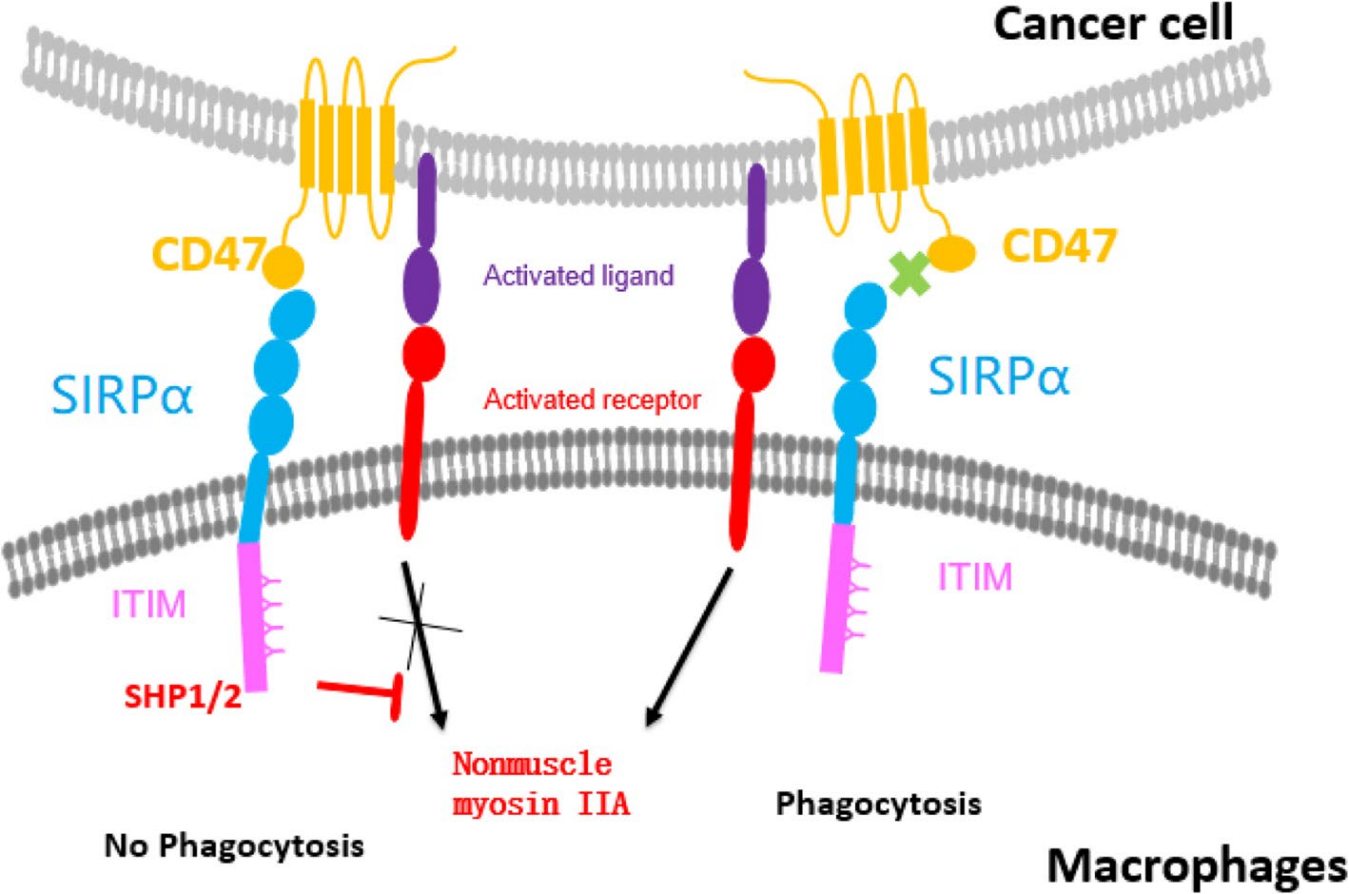
Biology of CD47/SIRPα interaction. CD47 binds to SIRPα to transmit inhibitory signals to macrophages and to inhibit or lessen phagocytic activity through uncoupling of receptor binding and signal transduction. Therapeutic agents block the interaction between CD47 and SIRPα to remove the inhibitory signal and restore the phagocytic activity

The interaction of the NH_2_-terminal IgV domain of CD47 with the D1 region of SIRPα promotes phosphorylation of tyrosine residues (Fig. 2). The phosphorylated ITIM recruits and activates protein tyrosine phosphatases (PTPase), including the Src homology region 2 (SH2)-domain-containing phosphatase-1 (SHP-1) and 2 (SHP-2). The interaction of the phosphatase SH2-domains with the phosphorylated ITIM of SIRPα disrupts its auto-inhibitory activity towards the PTPase domain, resulting in enzymatic activity. Dephosphorylation of phosphotyrosine residues on a variety of proximal substrates counterbalances activation of signaling pathways that depend on tyrosine phosphorylation, thereby restricting phagocytic function. CD47 was first identified as a “marker of self” on murine red blood cells, and was shown to interact with SIRPα to prevent phagocytosis of red blood cells by macrophages in the spleen [19]. Studies have shown that CD47 is broadly expressed on many types of normal cells and tissues. A study by Jaiswal demonstrated that CD47 was upregulated on circulating hematopoietic stem cells and leukemia cells, which prevented phagocytosis of these cells [11]. Targeting CD47 with anti-CD47 antibodies stimulated macrophage phagocytosis of AML cells in vitro and showed therapeutic efficacy against AML in mouse models [20]. In addition, CD47 was also overexpressed on hematologic [19, 21–23] and solid [24–26] malignancies, and treatment with agents that block CD47-SIRPα interaction stimulated macrophage phagocytosis in vitro and anti-tumor responses in vivo.

### Therapeutic strategy

The critical role of the CD47/SIRPα axis in the innate immune response suggests that these two proteins may be attractive therapeutic targets. Antagonists targeting the innate immune checkpoint CD47/SIRPα pathway are currently in clinical development. These antagonists include 1) monoclonal antibodies targeting CD47 or SIRPα, 2) SIRPα-Fc fusion proteins, 3) bispecific antibodies (BsAb), 4) small molecules to down-regulate CD47 on tumor cells, 5) RNAi and, 6) CD47-chimeric antigen receptor-T cell/Macrophages.

### Monoclonal antibodies and fc fusion proteins

**Fig. 3:**
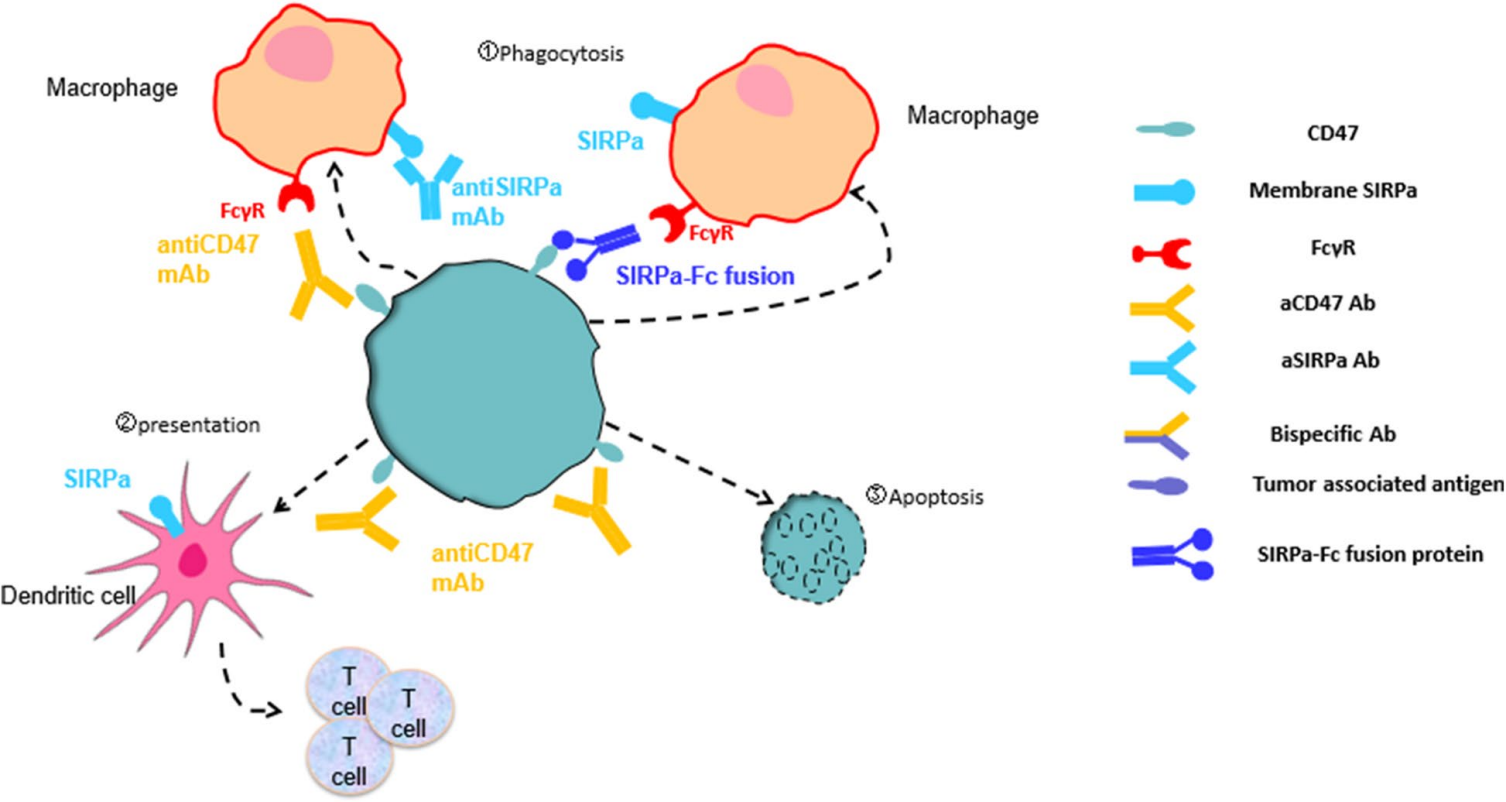
Mechanism of action of interruption of the CD47/SIRPα axis. Three mechanisms can be used to inhibit the CD47/SIRPα interaction. Phagocytosis (the most important mechanism of action): blocks the CD47/SIRPα interaction to remove inhibitory signals and promote phagocytosis of tumor cells; Antigen presentation: antiCD47 antibody connects tumor cells and SIRPα+ DCs to promote antigen presentation; Apoptosis: some antiCD47 antibodies could induce tumor cell poptosis

Three types of agents targeted to the CD47/SIRPα axis were developed: antibodies, SIRPα-Fc fusion proteins targeted to CD47, and antibodies targeted to SIRPα. The mechanisms of CD47-SIRPα blocking agents are summarized in Fig. 3. Agents targeted to CD47 should block the CD47-SIRPα interaction to remove the anti-phagocytic signal and restore the phagocytic activity of macrophages [27]. In addition, engagement of FcRs to limit activity is considered to be necessary for agents targeted to CD47 [28]. In addition, anti-SIRPα antibodies using inert Fc to prevent toxicity resulting from SIRPα expressed on myeloid immune cell perhaps have therapeutic potential.

The second therapeutic mechanism is bridging innate and acquired immunity [29]. Tumor cells are recognized, taken up by antigen presenting cells (APCs, such as dendritic cells and macrophages), and presented to naive T cells, resulting in T cell activation. Antibodies targeted to CD47 could induce direct killing of tumor cells by inhibiting protein kinase A via Giα, resulting in clustering of CD47 in the membrane, and caspase-independent programmed cell death [30].

#### Anti-CD47 monoclonal antibodies

More than ten anti-CD47 antibodies are in different phases of clinical development (Table 1). All of these antibodies are based on human IgG4-Fc, except AO-176, which is based on human IgG2-Fc [31]. Clinical study results of these antibodies were as follows (ordered by clinical trial developmental state).

**Table 1:** Summary of clinical trials with CD47-targeting antibodies

Antibody	Company	IgG subclass	Indications	Monotherapy or Combination	Phases stage	Clinical trial NO.	Status
Magrolimab (Hu5F9-G4)	Gilead Science (Forty-Seven)	Humanized, IgG4	Solid Tumor	monotherapy	Phase I	NCT02216409	88 participants, Completed
			AML, MDS	monotherapy	Phase I	NCT02678338	20 participants, Completed
			R/R B-cell NHL	Rituximab, Rituximab+Gemcitabine+Oxaliplatin	Phase I/II	NCT02953509	178 participants, Active, Not Recruiting
			Colorectal Neoplasms	Cetuximab	PhaseI	NCT02953782	78 participants, Completed
			AML, MDS	Monotherapy, Azacitidine	Phase I	NCT03248479	287 participants, Active Not Recruiting
			NHL, DLBC	Acalabrutinib and rituximab	Phase I	NCT03527147	30 participants, Completed
			Ovarian cancer	Avelumab	Phase I	NCT03558139	34 participants, Completed
			Urothelial carcinoma	Atezolizumab, Venetoclax	Phase I/II	NCT03869190	645 participants, Recruiting
			R/R AML	Atezolizumab	Phase I	NCT03922477	13 participants, Terminated
			R/R AML	Azacitidine and/or Venetoclax	Phase I/II	NCT04435691	98 participants, Recruiting
			R/R Indolent B-cell Malignancies	Obinutuzumab and Venetoclax	Phase I	NCT04599634	76 participants, Suspended
			TP53 mutant AML	Azacitidine and/or Venetoclax	Phase III	NCT04778397	346 particiants,Not recruiting
			R/R cHL	Pembrolizumab	Phase II	NCT04788043	24, participants, Not yet recruiting
			T cell lymphoma	Mogamulizumab	Phase I/II	NCT04541017	18 participants, Suspended
			Neuroblastoma or Relapsed Osteosarcoma	Dinutuximab	Phase I	NCT04751383	82 participants, Recruiting
Ligufalimab (AK117)	Akesobio Biopharma	Humanized, IgG4	Neoplasms Malignant	monotherapy	Phase I	NCT04349969	159 participants, Recruiting
			Neoplasms Malignant	monotherapy	Phase I	NCT04728334	162 participants, Recruiting
			MDS	Azacitidine	Phase I/II	NCT04900350	190 participants, Recruiting
			AML	Azacitidine	Phase Ib/II	NCT04980885	160 participants, Recruiting
			Advanced Malignant Tumors	AK112 + chemotherapy	Phase Ib/II	NCT05214482	160 participants, Recruiting
			Locally Advanced and Metastatic TNBC	AK112 ± Nab-Paclitaxel/ Paclitaxel)	Phase I	NCT05227664	80 participants, Not yet recruiting
			Advanced Malignant Tumors	AK112 ± (Caboplatin+Cisplatin+ 5-Fluorouracil)	Phase Ib/II	NCT05229497	114 participants, Not yet recruiting
			Advanced Malignant Tumors	AK104 + Oxaliplatin±Cisplatin±Paclitaxel±5-FU	Phase Ib/II	NCT05235542	130 participants, Not yet recruiting
Lemzoparlimab (TJC4)	I-Mab BIOPARMA	Human, IgG4	Solid tumors, lymphoma	Pembrolizumab and Rituximab	Phase I	NCT03934814	116 participants, Recruiting
			AML, MDS	monotherapy	Phase I/II	NCT04202003	80 participants, Recruiting
			Multiple myeloma	Dexamethasone+Pomalidomide±Carfilzomib, Dexamethasone+Pomalidomide+Daratumumab	Phase Ib	NCT04895410	163 participants, Recruiting
			AML, MDS	Azacitidine±Venetoclax	Phase Ib	NCT04912063	80 participants, Recruiting
			Advanced Solid Tumor	toripalimab	Phase I/II	NCT05148533	96 participants, Recruiting
AO-176	Arch Oncology	Humanized, IgG2	Solid Tumor	Monotherapy,+Paclitaxel, Pembrolizumab	Phase I/II	NCT03834948	183 participants, Recruiting
			R/R Multiple Myeloma	Dexamethason±Bortezomib	Phase I/II	NCT04445701	102 participants, Recruiting
CC-90002	Celgene (Inhibrx)-BMS	Humanized, IgG4	Hematologic Neoplasms	monotherapy, Rituximab	Phase I	NCT02367196	60 participants, Completed
			Leukemia, Myeloid, AMDS	monotherapy	Phase I	NCT02641002	28 participants, Terminated
SGN-CD47M	Seagen Inc.	Humanized, IgG4	solid tumor	monotherapy	Phase I	NCT03957096	16 participants, Terminated
IBI188	Innovent Biologics, Inc.	Human, IgG4	Advanced Malignancies	monotherapy, Rituximab	Phase Ia	NCT03717103	49 participants, Active, not recruiting
			Advanced Malignancies	monotherapy	Phase I	NCT03763149	20 participants, Recruiting
			AML	azacitidine	Phase Ib	NCT04485052	126 participants, Recruiting
			Higher Risk MDS	azacitidine	Phase I	NCT04485065	12 participants, Recruiting
			Solid Tumors, Lung Adenocarcinoma, Osteosarcoma	Sintilimab, Cisplatin/Carboplatin+Bevacizumab+Pemetrexed, GM-CSF	Phase Ib	NCT04861948	120 participants, Recruiting
			MDS	Azacitidine	Phase Ib	NCT04511975	32 participants, Suspended
SHR-1603	HENGRUI Medicine	Human, IgG4	Advanced Cancer	monotherapy	Phase I	NCT03722186	128 participants, Suspended (Business Decision)
SRF231	Surface Oncology	Human IgG4	Advanced Solid Cancers, Hematologic Cancers	monotherapy	Phase I/Ib	NCT03512340	148 participants, Completed
ZL-1201	ZAI lab	Humanized, IgG4	Locally Advanced Solid Tumor	monotherapy	Phase I	NCT04257617	66 participants, Recruiting
IMC-002	ImmuneOncia Therapeutics	Human	Solid Tumor, Lymphoma	monotherapy	Phase I	NCT04306224	24 participants, Recruiting
			Advanced Cancer	monotherapy	Phase I	NCT05276310	24 participants, Not yet recruiting

##### Magrolimab (Hu5F9-G4)

Magrolimab, previously known as Hu5F9-G4, was the first anti-CD47 antibody to enter clinical trials, and is currently in Phase III development. The clinical trials for magrolimab are listed in the Table 1. In a preclinical study [32], combined treatment with magrolimab and rituximab showed significant clearance of Raji cells in vitro and elimination of AML tumor cells in vivo. Magrolimab blocks the CD47/SIRPα interaction and the anti-phagocytic signal on macrophages, and binding of rituximab to FcRs initiates pro-phagocytic signaling. Activation of pro-phagocytic signaling is a beneficial effect of anti-CD47 antibody treatment. Magrolimab caused significant hemagglutination and phagocytosis of RBCs in vitro, which indicated potential toxicity. Non-human primate pharmacokinetic and toxicology studies showed dose-dependent anemia. To overcome treatment-related anemia and thrombocytopenia, a low priming dose was given to stimulate production of new RBCs and to facilitate tolerance of subsequent higher maintenance doses. The same strategy (1 mg/kg priming dose on day 1) effectively controlled anemia during subsequent infusion of magrolimab in a clinical trial. The saturation concentration (receptor occupancy) on circulating white and red cells was 30 mg/kg [33]. In a phase Ib [34] (NCT02953509) study evaluating relapsed or refractory non-Hodgkin’s lymphoma, patients with diffuse large B-cell lymphoma (DLBCL) or follicular lymphoma were treated with magrolimab in combination with rituximab. The results showed that 50% of the patients had an objective (i.e., complete or partial) response, with 36% having a complete response. Specifically, the rates of objective response and complete response were 40 and 33%, respectively, among patients with DLBCL, and 71 and 43%, respectively, among those with follicular lymphoma. Calreticulin (CRT) is a member of the endoplasmic reticulum lectin chaperone family of proteins that plays important biological roles in Ca^2+^ homeostasis (in the endoplasmic reticulum, ER), integrin-dependent cell adhesion (in the cytoplasm), and immune response activation (on the plasma membrane) [35]. CRT translocates from the ER to the cell surface during immunogenic cell death in response to various stress factors such as chemotherapy, irradiation, photodynamic therapy, and cytokines. CRT on the surface of stressed or dying cells acts as an “eat me” signal by binding to LRP1 on macrophages [36].

Azacitidine is a chemotherapeutic drug that could induce ICD through translocation of CRT to the cell surface. Interaction of CRT with LRP1 transmits “eat me” signals to macrophages and promotes phagocytosis of dying cells [37]. Combined treatment with magrolimab and azacitidine in a preclinical study resulted in significantly increased phagocytic activity in vitro, and elimination of HL60 in vivo [38]. Preliminary data from a trial (NCT04778397) of magrolimab combined with azacitidine for treatment of AML resulted in a 65% OR and a 40% CR in 34 patients. Transient on-target anemia was observed, and 56% of patients with AML became red blood cell transfusion independent in response to this therapy. In patients with TP53-mutant AML, 15/21 (71%) achieved an OR and 10 (48%) achieved CR [39]. However, this study was halted by the FDA due to imbalance in investigator-reported suspected unexpected serious adverse reactions (SUSARs). Both magrolimab [33] and azacitidine [40] induce anaemia, neutropenia and thrombocytopenia. The combination of magrolimab and azacidine may increase hematological toxicity.

##### Ligufalimab (AK117)

Ligufalimab (AK117) is a humanized IgG4 antibody against CD47 from Akeso Biopharma that has completed a phase I trial in Australia, and is currently in phase II trials in China and Australia. In pre-clinical studies, Ligufalimab was comparable to magrolimab with regard to EC50 values and induction of phagocytic activity on Raji and Jurkat cells. However, Ligufalimab did not induce hemagglutination at concentrations up to 1050 μg/ml, while magrolimab induced strong hemagglutination at 1.44 μg/ml (unpublished data by our lab). In a clinical trial (NCT04349969) [41] evaluating treatment of R/R advanced or metastatic solid or lymphoid tumors, Ligufalimab did not induce symptoms of drug-related anemia at doses up to 45 mg/kg, and no priming dose was required. A CD47 receptor binding study on peripheral T cells in patients dosed with 3 mg/kg of Ligufalimab showed nearly 100% binding. No Dose-limiting toxicity (DLTs) occurred in subjects receiving up to 45 mg/kg QW (*quaque* week) of Ligufalimab. Combination with bispecific antibody AK104 (PD1-CTLA4), AK112 (PD1-VEGF).

##### Lemzoparlimab (TJC4)

Lemzoparlimab (TJC4), from I-MAB, is a human IgG4 antibody targeted to CD47 that was screened using a phage display system. The crystal structure of the TJC4/CD47 complex (straighter head-to-head orientation) showed that TJC4 binds to a different epitope than does magrolimab (tilted head-to-head orientation) [42]. In addition, TJC4 showed minimal binding to RBC and no hemagglutination was observed at 100 μg/ml in vitro. No significant erythrocyte toxicity was observed in cynomolgus monkeys dosed with 10–100 mg/kg QW. A phase I trial (NCT03934814) [43] evaluating treatment of R/R advanced solid tumors and lymphoma with TJC4 alone, or in combination with pembrolizumab or rituximab, is ongoing. No DLT or SAE (severe adverse events) were observed, and treatment-associated anemia occurred in 30% of patients (6 of 20, 2 receiving 3 mg/kg, 2 receiving 10 mg/kg, 1 receiving 20 mg/kg, and 1 receiving 30 mg/kg). Anti-drug antibody (ADA) events occurred in 25% of patients, but there were no concerns regarding safety or pharmacokinetics (PK). Maximal saturation of peripheral T cells was achieved at a dose of 20 mg/kg administered weekly.

##### AO176

AO176, developed by Arch Oncology, is a humanized IgG2 subclass anti-CD47 antibody [31]. AO176 was shown to bind to integrin-β1 expressed on tumor cells, but not on RBC. Interestingly, AO176 blocked the CD47/SIRPα interaction to stimulate phagocytosis of tumor cells, and also directly killed tumor cells (non-ADCC). A tolerability and hematologic study on Cynomolgus monkeys showed no anemia. In phase I/II clinical trials [44], Grade 3 TRAE (treatment-related adverse events) were observed in > 10% of patients. In addition, DLT was observed at 20 mg/kg. Studies evaluating AO176 monotherapy and combination therapy with paclitaxel are ongoing.

##### CC-90002

CC-90002 is a humanized monoclonal IgG4 CD47 antibody. An early clinical trial (NCT02641002) that evaluated treatment of Acute Myeloid Leukemia (AML) and high-risk myelodysplastic syndrome (MDS) was terminated due to poor activity and safety profiles [45, 46]. Another phase I trial (NCT02367196) evaluated treatment of advanced solid (alone) and hematologic malignancies (in combination with rituximab). In a combination trial with patients with R/R NHL [47], the ORR (overall response rate) was 13% with 25% achieving stable disease. However, 50% had anemia (of any grade), 33% had thrombocytopenia, and DLTs were observed in 3 subjects (1 subject infusion-related reaction at 15 mg/kg Q2W and 2 subjects had grade 3 thrombocytopenia requiring platelet transfusion occurring at 30 mg/kg).

##### SGN-CD47M

SGN-CD47M, developed by Seagen (Seattle Genetics), is a CD47 targeting probody drug conjugate (PDCs). Probody therapeutics [48] are antibody prodrugs designed to remain inactive until proteolytically cleaved and activated in the tumor microenvironment. Probody drug conjugates [49] can be activated by multiple proteases in the tumor microenvironment, but remain inactive in the circulation and in normal tissues. The efficacy of PDCs depends on multiple factors including binding affinity and specificity for the antigen, efficiency of cleavage in the tumor microenvironment, lack of cleavage in normal tissues, and internalization efficiency. Studies focused on PDCs are in the early clinical stage, and safety and efficacy have yet to be determined. Clinical trial (NCT03957096) of SGN-CD47M for treatment of advanced solid tumors was terminated based on portfolio prioritization.

Other drugs in early clinical development for safety and dosing evaluation are listed in Table 1.

##### SIRPα-fc

Among the 10 allelic variants of SIRPα, SIRPα V1 and V2 are the most prevalent variants [50]. The affinity of wild type SIRPα binding to CD47 is in the micromolar range, which is 1000-fold weaker than that of anti-CD47 antibodies. Soluble SIRPα binding to CD47 on tumor cells could block inhibitory signals and enhance phagocytic activity. Six SIRPα-fusion proteins are currently in phase I or phase II clinical trials (Table 2, ordered by clinical trial developmental state).

**Table 2:** Summary of clinical trials targeting CD47 with SIRPα-Fc fusion protein

SIRPα-Fc fusion	Company	IgG	Indications	Monotherapy or Combination	Phage stage	Clinical trials NO.	Status
Evorpacept (ALX148)	Alex Therapeutic	mutated SIRPα-Fc IgG1	Metastatic Cancer, Solid Tumor, Advanced Cancer, NHL	monotherapy, Pembrolizumab, Trastuzumab, rituximab, Pembrolizumab+ 5-FU + Cisplatin, Trastuzumab+Ramucirumab+Paclitaxel	Phase I	NCT03013218	174 participants, Active, not recruiting
			Higher Risk Myelodysplastic Syndrome	Azacitidine	Phase I	NCT04417517	173 participants, Recruiting
			HNSCC	Pembrolizumab	Phase II	NCT04675294	183 participants, Recruiting
			HNSCC	Pembrolizumab+Cisplatin/Carboplatin+5FU	Phase II	NCT04675333	168 participants,
			AML	Venetoclax, Azacitidine	Phase I/II	NCT04755244	97 participants, Recruiting
			HER2+ gastric cancer	Trastuzumab, Ramucirumab, Paclitaxel	Phase II/III	NCT05002127	450 participants, Recruiting
			Indolent and Aggressive B-Cell Non-Hodgkin Lymphoma	Lenalidomide+Rituximab	Phase I/II	NCT05025800	52 participants, Recruiting
			HER2+ gastric cancer	Zanidatamab	Phase Ib/II	NCT05027139	93 participants, Recruiting
			Microsatellite Stable Metastatic Colorectal Cancer	Cetuximab+Pembrolizumab	Phase II	NCT05167409	80 participants, Not yet recruiting
TTI-621	Trillium Therapeutics	SIRPα-Fc IgG1	Hematologic Malignancies and Selected Solid Tumors	monotherapy, Rituximab and Nivolumab	Phase I	NCT02663518	260 participants, Recruiting
			R/R Solid Tumors and Mycosis Fungoides	monotherapy, PD-1/PD-L1 Inhibitor, pegylated interferon-α2a, T-Vec, radiation	Phase I	NCT02890368	56 participants, terrminated
			Leiomyosarcoma	Doxorubicin	Phase I/II	NCT04996004	80 participants, Recruiting
			Multiple Myeloma	Daratumumab Hyaluronidase-fihj	Phase I	NCT05139225	40 participants, Recruiting
TTI-622		SIRPα-Fc IgG4	Lymphoma, myeloma	monotherapy, Rituximab, PD-1 Inhibitor, Proteasome-inhibitor Regimen	Phase I	NCT03530683	150 participants, Recruiting
			Multiple Myeloma	Daratumumab Hyaluronidase-fihj	Phase I	NCT05139225	40 participants, Recruiting
			Platinum-Resistant Ovarian Cancer	Pegylated Liposomal Doxorubicin	Phase I/II	NCT05261490	50 participants, Recruiting
IMM01	ImmunoOnco	mutated SIRPα-Fc IgG1	AML, MDS	Azacitidine	Phase I/II	NCT05140811	76 participants, Not yet recruiting

##### Evorpacept (ALX148)

Evorpacept is comprised of SIRPα variant 1 domain 1 (V1D1) and inactive human IgG1-Fc [51]. Evorpacept (CV1) was selected from mutant libraries and includes 9 mutations (V6I, A271, I31F, E47V, K53R, E54S, H56P, L66T, V92I), which resulted in a 50,000-fold increase in affinity compared with that of wild type SIRPα. Preclinical data showed that evorpacept augmented macrophage antitumor activity in vitro and in vivo in combination with tumor-opsonizing antibodies (trastuzumab, obinutuzumab and cetuximab). However, no phagocytosis or antitumor activity was observed following treatment with evorpacept alone. A trial [52] in which patients with NHL received evorpacept alone at 10 mg/kg or at 15 mg/kg in combination with rituximab resulted in ORRs of 40.9 and 63.6%, respectively. The CD47 receptor occupancy on RBC and CD4 T cells was approximately 90% at 10–15 mg/kg. A phase I study [53] of evorpacept in combination with pembrolizumab, trastuzumab, or zanidatamab, and/or chemotherapeutic agents, evaluating treatment of advanced solid malignancy is ongoing. Preliminary results showed anti-cancer activity of evorpacept in combination with pembrolizumab (AP) and/or chemotherapy (5FU + platinum) in patients with second line or greater HNSCC (head and neck squamous cell carcinoma) with prior platinum therapy. The ORR in patients with checkpoint inhibitor-naïve HNSCC (*n* = 10) treated with AP was 40%, but 0% in patients with HNSCC who had previously received checkpoint inhibitors (*n* = 10). A phase II study of evorpacept in combination with pembrolizumab for treatment of HNSCC was recently initiated. Treatment with evorpacept in combination with transtuzumab, ramucirumab, and paclitaxel (TRP) showed favorable tolerability and demonstrated objective response in patients with HER2-positive gastric/gastroesophageal cancer. The maximum tolerated dose was not reached in this study. The maximum administered dose was15 mg/kg QW with 22.2% grade 3 or above TARE. The ORR of evorpacept (10 or 15 mg/kg, QW) in combination with TRP in patients with second line HER2 positive gastric/GEJ (gastroesophageal junction) cancer was 72.2%.

##### TTI-621 and TTI-622

TT1–621 and TT1–622 are constructed from the wild type SIRPα variant 2 domain 1 (V2D1) fused to human IgG-Fc with IgG1 and IgG4 backbones, respectively. Preclinical data demonstrated binding of TTI-621/TTI-622 to cancer cells but only minimal binding to RBCs [54]. However, TTI-621 caused significant anemia in monkeys, presumably due to activation of NK cells by wild type IgG1 with intact Fc function. Both molecules augment tumor-cell killing mediated by macrophages and T cells (phagocytosis by macrophages and presentation of tumor antigens to CD8 T cells to stimulate cytotoxicity, respectively) and both exhibited enhanced activity when used in combination in a preclinical study. Monotherapy using TTI-621 (NCT02663518) or TTI-622 (NCT03530683) against R/R lymphoma induced transient anemia and thrombocytopenia, with recovery within 7 days. The CD47 receptor occupancy on normal peripheral T cells was 60% at a dose of 2 mg/kg. In addition, TTI-622 was well tolerated at 18 mg/kg and the ORR of TTI-622 during treatment of R/R lymphomas was 33%. The ORR for TTI-621 was 18–29% at up to 2 mg/kg and two dosing levels (0.2 and 2.0 mg/kg) will be evaluated in phase Ib/II [55].

##### IMM01

IMM01 is V2D1 with an N80A mutation and IMM0306 is IMM01-fused to an IgG1 anti-CD20 antibody (rituximab). Both IMM01 and IMM0306 are based on wild type IgG1-Fc. IMM01 does not bind to human erythrocytes, avoiding “antigenic sink.” Monotherapy using IMM01 was administered to 14 patients with R/R lymphoma [56]. Transient platelet count decreased after 2 h and returned to baseline at 24 to 48 h following the first infusion. One patient experienced grade 3 platelet count decrease. Preliminary results showed anti-tumor activity at 1.0 mg/kg. In preclinical in vivo study, IMM01 showed strong synergistic anti-tumor activity combined with rituximab (antiCD20 antibody), imatinib (tyrosine kinase inhibitor, TKI). Clinical trials are ongoing of IMM01 combined with rituximab, imatinib in China.

##### HX009

HX009 (Table 3) is wild type SIRPα V2D1 fused to the C terminal of an IgG4 anti-PD1 antibody. A phase I clinical trial (NCT04097769) showed that HX009 was well-tolerated at 7.5 mg/kg with no DLT [57] when used to treat patients with advanced solid tumors. Antitumor activity was seen at 1 mg/kg and 5 mg/kg cohorts with objective responses in multiple tumor types (gallbladder adenocarcinoma (1 mg/kg), triple negative breast cancer (5 mg/kg), metastatic squamous cell carcinoma of head and neck (5 mg/kg)). Phase I/II trial in Chinese patients with relapsed/refractory lymphoma is ongoing.

**Table 3:** Bispecific antibodies targeting CD47 and other molecular targets

Code	Company/Research Team	Target	Structure	IgG subclass	Format
NI-1701 (TG-1801)^a^	LightChain bioscience/TG Therapeutics	CD47 × CD19	Fab+Fab with Fc, 1 + 1, κλ	IgG1	one antibody arm (kappa) specific for CD47 and a second arm (lambda) specific to CD19, mesothelin or tumor associated antigen
IMM-0306^a^	ImmuneOnco Biopharma	CD47 × CD20	ligand+Fab with Fc, 2 + 2	IgG1	SIRPα V2-D1 infused with amino terminal of heavy chain of Rituximab
DVD-Ig SL/LL	Ravindra Majeti’s lab in Stanford University	CD47 × CD20	DVD-IgG	IgG1	The amino terminus of each B6H12 variable domain infused to the carboxyl terminus of each 2B8 with linker”TVAAP”
SIRPa-gamma-CD20 HC		CD47 × CD20	ligand+IgG	IgG1	ligand located in Fab domain of intact aCD20 antibody(2B8, Rituximab)
CD20-2GL-SIRPa HC; CD20-4GL-SIRPa HC		CD47 × CD20	ligand+IgG	IgG1	ligand located in Fc domain of intact aCD20 antibody(2B8, Rituximab)
bi-scFv RTX-CD47	Wijnand Helfrich’s lab in Wijnand Helfrich	CD47 × CD20	ScFV+ScFv,1 + 1	NA	CD20-targeting scFv antibody fragment derived from rituximab fused in tandem to a CD47-blocking scFv
LQ007	Novamab	CD47 × CD20	nanobody (C terminal) + IgG	IgG4	nanobody from camel was fused to the carboxyl terminus of Rituximab
HMBD-004A	Hummingbird bioscience	CD47 × CD33	Fab+Fab with Fc, 1 + 1	IgG1	Humanized anti-CD47 variable domain as an effector arm and the gemtuzumab variable domain as an anti-CD33 or BCMA specificity arm.
HMBD-004B	Hummingbird bioscience	CD47 × BCMA	Fab+Fab with Fc, 1 + 1	IgG1	
NI-1801^a^	Light Chain bioscience (Novimmune SA)	CD47 × MSLN	Fab+Fab with Fc,1 + 1,κλ	IgG1	one arm target to CD47(kappa) and the other arm target to (lambda) mesothelin or tumor associated antigen
NI-2401	Light Chain bioscience (Novimmune SA)	CD47 × TAA	Fab+Fab with Fc,1 + 1,κλ	Not Disclosed	
NI-2601	Light Chain bioscience (Novimmune SA)	CD47 × TAA	Fab+Fab with Fc,1 + 1,κλ	Not Disclosed	
PT-886	Phanes Therapeutics	CD47 × CLDN18.2	Fab+Fab with Fc, 1 + 1	Not Disclosed	Not Disclosed
PT-796	Phanes Therapeutics	CD47 × FRα	Fab+Fab with Fc, 1 + 1	Not Disclosed	Not Disclosed
PT-217	Phanes Therapeutics	CD47 × DLL3	Fab+Fab with Fc, 1 + 1	Not Disclosed	Not Disclosed
IMM-26011	ImmuneOnco Biopharma	CD47 × FLT-3	ligand+Fab with Fc, 2 + 2	IgG1	SIRPa V2-D1 linked
IMM-2902	ImmuneOnco Biopharma	CD47 × Her2	ligand+Fab with Fc,2 + 2	IgG1	SIRPa V2-D1 linked through a GS linker to amino terminal of light chain coding sequence of Herceptin (Trastuzumab)
SG3847	SUMGEN BIOTECH	CD47 × CD38	ligand+Fab with Fc, 2 + 2	IgG1	SIRPa V1-D1 linked through a GS linker to amino terminal of light chain coding sequence of
HX-009^a^	*HanX Biopharmaceuticals*	CD47 × PD-1	ligand +Fab with Fc, 2 + 2	IgG4	amino terminal of extracellular region ligand (SIRPA V2D1) infused with carboxyl terminus of anti PD1 antibody (HX008)
IBI-322^a^	Innovent	CD47 × PD-L1	Fab+nanobody with Fc,1 + 2	Not Disclosed	one arm target CD47 (Fab from IBI188), the other target is PDL1 (Bivalent nanobody)
BH-29XX	Hanmi Pharmaceutical	CD47 × PD-L1	Fab+Fab with Fc, 1 + 1	Not Disclosed	Not Disclosed
?	GenSci	CD47 × PD-L1	Fab+Fv, KIH, 1 + 1	Not Disclosed	one arm target CD47 (Fab from GenSci-059), the other target is PDL-1(Fv from GenSci-047)
PMC-122	PharmAbcine	CD47 × PD-L1	Not Disclosed	Not Disclosed	Unknown
ABP-160	Abpro Therapeutics	CD47 × PD-L1	Not Disclosed	IgG1 wt	Unknown
IMM-2505	ImmuneOnco Biopharma	CD47 × PD-L1	ligand+Fab with Fc, 2 + 2	IgG1	SIRPa V2-D1 infused with amino terminal of heavy chain of antiPDL-1 antibody (Atezolizumab?)
TJ-L1C4	I-MAB Biopharma	CD47 × PD-L1	Not Disclosed	Not Disclosed	PD-L1 antibody acts as the backbone and is linked with CD47 antibody
IAB	Sinomab	CD47× PD-L1	CV1(ALX148) + Fab with Fc	IgG1/IgG4	variable region of atezolizumab and consensus variant 1 (CV1) monomer
SL-172154^a^	Shattuck Labs	CD47× CD40	ligand+ligand with Fc, 1 + 1	IgG1	ECDs of SIRPα and CD 40 L, adjoined by a central domain termed SIRPα-Fc-CD 40 L.
DSP107	Kahr Medical	CD47 × 41BB	ligand+ligand with Fc, 1 + 1	IgG1	human soluble SIRPα and 4-1BBL
TJ C4GM	I-MAB Biopharma	CD47 × CSF-2R	IgG + fusion protein	IgG1 or G4	Human GM-CSF was fused to the heavy chain C terminus of CD47 antibody
IMM-0207	ImmuneOnco Biopharma	CD47× VEGF	ligand+receptor with Fc, 2 + 2	IgG1	SIRPa V2-D1 linked via an Fc fragment of an Ig to an Ig region of an extracellular domain of VEGFR1D

^a^antibody in clinical trial

#### Antibodies targeting SIRPα on myeloid cells

Anti-SIRPα antibodies induce weak or no phagocytic activity alone, but induce significantly increased phagocytic activity when combined with opsonizing antibodies (rituximab, cetuximab) [58–60]. Several issues with anti-SIRPα antibodies are important to consider. First, since SIRPα is expressed on myeloid cells, anti-SIRPα antibodies using inactive human IgG-Fc to avoid Fc effector-mediated toxicity on these immune cells may be advantageous [60]. Second, SIRPγ expressed on T and NK cell shares 74.37% amino acid similarity with the extracellular domain with SIRPα [61]. SIRPγ on T cells binds to CD47 on APCs to mediate cell-cell adhesion and enhances antigen presentation, resulting in T cell proliferation and cytokine secretion [62]. Development of a SIRPα targeting antibody with specificity toward SIRPα to avoid interference with the interaction between CD47 and SIRPγ may preserve T cell activity. Third, antibody internalization could lead to rapid clearance of antibody in vivo. Higher doses or multiple dosing is required to ensure that levels remain therapeutically relevant [63, 64]. Internalization of SIRPα decreases the inhibitory signal and may enhance the ability of antibodies to restore phagocytic activity [59, 65]. Finally, Although SIRPα has ten known variants, V1, V2, and V8 are the most prominent (over 90%) haplotypes in the human population. Antibodies targeting all three of these variants may be more potent than those that target a single variant [66, 67].

Antibodies targeting SIRPα currently being evaluated are listed in the Table 4. Trials evaluating treatment with SIRPα antibodies in combination with immunotherapies are in the early clinical stage of development, and include OSE-172 [68] from OSE Immunotherapeutics (co-developed with Boehringer Ingelheim), CC-95251 from Celgene, and FSI-189 from Gilead [59].

**Table 4:** Summary of antibodies targeting SIRPα on myeloid cells

Code	Company	IgG	Indications	Monotherapy and Combination	Clinical trials NO.	Status
OSE-172 (BI 765063)	OSE Immuno therapeutics & Boehringer Ingelheim	Humanized IgG4	Solid Tumor	monotherapy, BI 754091(antiPD1 Abs)	NCT03990233	116 participants, Recruiting
			Solid Tumor	antiPD1 Abs (BI 754091)	NCT04653142	18 participants, Active not recruiting
			NSCLC, HNSCC, Melanoma	Ezabenlimab	NCT05068102	22 participants, Recruiting
			HNSCC	Ezabenlimab+Cetuximab, Chemotherapy/BI754091(antiPD1 Abs)/BI 836880 (antiVEGF)	NCT05249426	150 participants, Recruiting
CC-95251	Celgene	Human	Advanced Solid and Hematologic Cancers	monotherapy, Rituximab, Cetuximab	NCT03783403	230 participants, Recruiting
			AML, MDS	Azacitidine	NCT05168202	30 participants, Recruiting
FSI-189	Gilead Sciences	Humanized inert IgG1	R/R NHL	Rituximab	NCT04502706	9 participants, Not yet recruiting
HSIRPB	Arch Oncology	Not disclosed	PRECLINICAL	NA	NA	NA
H21	ALX Oncology	Not disclosed	PRECLINICAL	NA	NA	NA
ES004	ElpiScience	Not disclosed	PRECLINICAL	NA	NA	NA
AL008	Alector/Innovent	IgG4	PRECLINICAL	NA	NA	NA
ADU-1805	Aduro Biotech	Humanized IgG2	PRECLINICAL	NA	NA	NA
Abx701	Apexigen		PRECLINICAL	NA	NA	NA

*NA* not applicable

### Bi-specific molecules

Bi-specific molecules bind two targets or two distinct epitopes of one target. The antigen binding sites of bi-specific molecules could consist of two antibodies or proteins (ligand or receptor), or could consist of one antibody and one protein. Bi-specific antibodies can bind two target antigens *in-cis* and *in-trans*. Rational design of bi-specific molecules based on biological activity may result in distinct effects or improved efficacy when compared to combination treatments. Four bispecific antibodies, catumaxomab [69] (withdrawn in 2017), blinatumomab [70], emicizumab [71], and amivantamab-vmjw [72] have been approved by EMA or FDA.

**Fig. 4:**
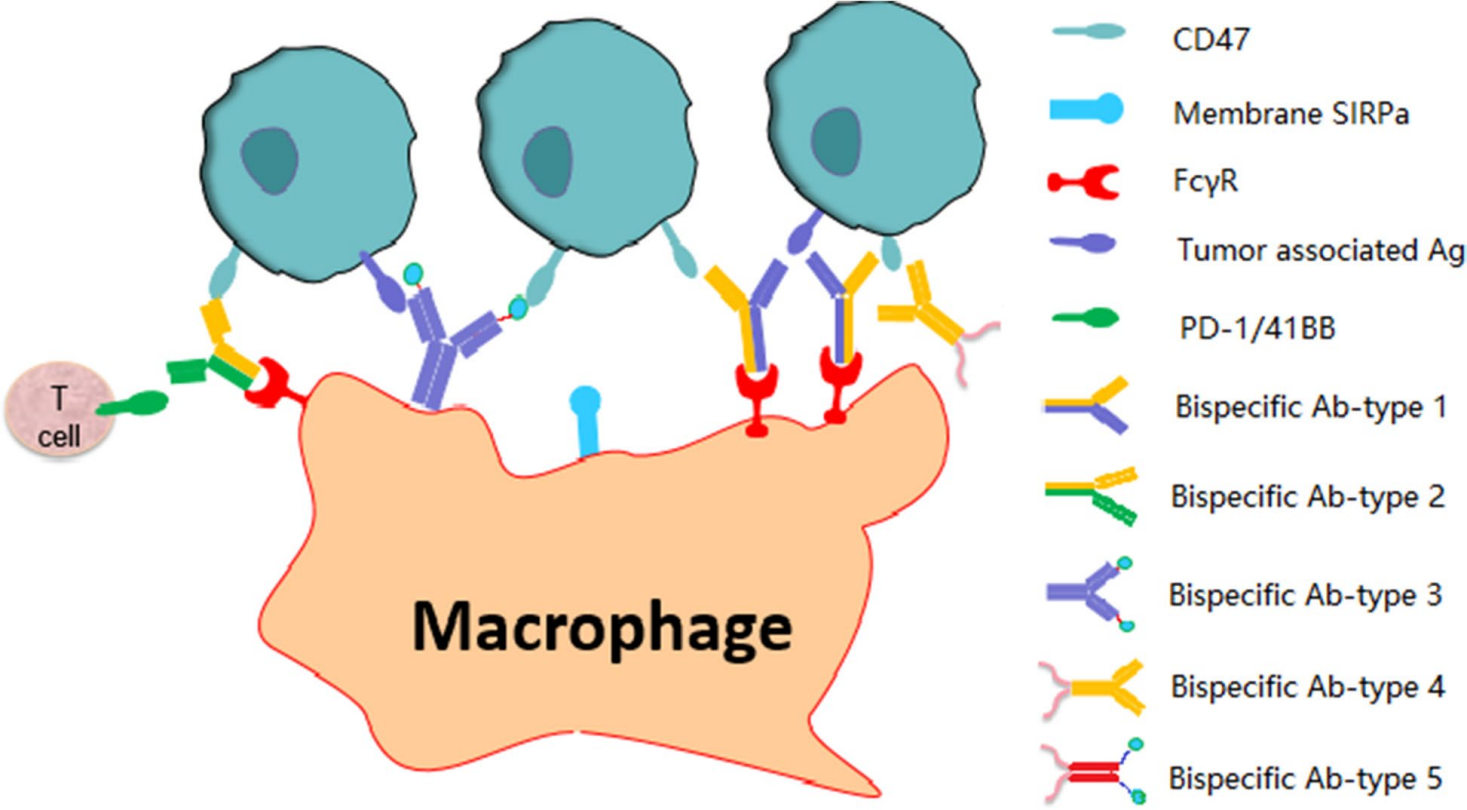
Types of bis-specific molecules targeted CD47 and other molecules. Bispecific Ab-type 1: KIH format, targeted to CD47 (yellow) and other tumor associated antigens (CD19, CD20, and MSLN, blue); Bi-specific Ab-type 2: KIH format, targeted to CD47 (yellow) and immune checkpoint molecules (PD-1, CD40, and 41BB, green); Bi-specific Ab-type 3: SIRPα (cyan) fusion to N-terminus of H-chain of IgG (targeting tumor associated antigen, blue); Bi-specific Ab-type 4: ligand/receptor to modulate TME (GM-CSF, and VEGFR2, pink) fusion to the C-terminus of the H-chain of IgG (yellow); Bi-specific Ab-type 4: SIRPα fusion to the N-terminus of IgG-Fc and ligand/receptor to modulate TME (GM-CSF, VEGFR2, pink) fusion to the C-terminus

Bi-specific molecules can be constructed from CD47 targeting antibodies or SIRPα and other antigen-targeting molecules (Fig. 4). Target antigens could include: A) tumor associated cell surface antigens (PD-L1, CD20, CD19, MSLN (Mesothelin), Claudin18.2, and Her2), B) immune checkpoint proteins (PD-1, CD40, 41BB), and C) cytokines or receptors (CSF-2 receptor, VEGF). Reduced affinity for CD47 and increased affinity for the second target may reduce toxicity and enhance efficacy. For type A antibodies, IgG1-Fc was selected to enhance antibody-mediated killing of tumor cells (ADCC, ADCP, and CDC). However, inactivated Fc is preferred when used in type B bi-specific molecules. Use of CD47-targeting biologics has shown significant clinical efficacy for treatment of R/R AML, NHL, and MDS. The combination of Hu5F9 and rituximab showed particular promise as a treatment approach for R/R NHL [34]. Several CD47-related BsAb are in early-stage clinical trials. NI1701 [73], which targets CD47 and CD19, is an IgG-like BsAb constructed using modified knobs-into-hole technology [74], and contains an IgG1-Fc. Preclinical data showed that NI1701 [75] selectively binds to CD47 and CD19 co-expressing cells, but interacts poorly with normal healthy cells (CD47^+^CD19^−^), resulting in avoidance of normal cells acting as sinks for binding of the antibodies, and reduced toxicity. In vitro and in vivo studies showed that NI1701 more potently killed tumor cells than did anti-CD47 and anti-CD19 antibodies alone, or in combination. A BsAb named NI1801 [73] targeted to CD47 and MSLN showed similar preclinical activity as NI1701. IMM0306, a bispecific antibody fusion protein targeted to CD20 (rituximab) and CD47 (SIRPα) with wild type IgG1-Fc [76], is in a phase I trial evaluating treatment of R/R CD20-positive B-cell non-Hodgkin’s lymphoma. SL-172154 [77] is a fusion protein targeted to CD47 with SIRPα and CD40 with CD40L, and is in a phase I trial for treatment of solid tumors. A BsAb, HX009, comprised of the extracellular region of SIRPα V2D fused with an anti-PD1 antibody (HX008) was evaluated in a phase I trial. The study showed that HX009 blocked both CD47/SIRPα and PD-1/PDL-1 interactions, and interacted with CD47 on tumor cells and PD1 on T cells to help present tumor antigens to T cells, resulting in activation of the innate and acquired immune responses. Several BsAbs have been developed to inhibit CD47/SIRPα and PD-1/PD-L1 interactions. One such BsAb, IBI322 [78], was designed as a selective CD47 binding CD47/PD-L1 bispecific antibody. IBI322 showed no negative hematological effects in cynomolgus monkeys, but the binding affinity of IBI322 to cynomolgus CD47 was not disclosed, and IBI322 showed dose-dependent binding to RBC. Other BsAb antibodies listed in Table 2 are in the preclinical proof-of-concept stage.

### Engineered T cells and macrophages

T cells with chimeric antigen receptors (CARs) showed promising therapeutic efficacy against hematologic malignancies, and several CAR-T therapeutics have been approved [79–83]. However, low treatment response rates of solid tumors to CAR-T treatment were observed. Golubovskaya et al. [84] showed that CD47-CAR-T cells effectively killed ovarian, pancreatic, and other cancer cells, and induced production of high levels of IL-2, which correlated with expression of CD47 antigens. Treatment with CD47-CAR-T cells may be a novel strategy for treating different types of cancers. Huyen [85] designed a third generation of CD47-CAR-T cell that could effectively kill lung cancer cells (A549) and inhibit lung cancer cell metastasis. A dual CAR-T targeting CD47 and TAG-72 (tumor-associated glycoprotein 72) generated by Shu [86] showed promising results against ovarian cancer in preclinical experiments. An Anti-PD-L1 (A12) CAR-T with the ability to secrete anti-CD47 VHH (variable heavy domain of heavy chain antibodies or nanobodies) (A4), developed by Xie [87], represented a novel strategy for cancer treatment. An A12-A4 CAR-T showed better anti-cancer activity than an A12 CAR-T plus soluble A4 in C57BL/6 PD-L1-KO mice bearing B16F10 cells. Each of these CD47-CAR-T cells are in the preclinical stage, and efficacy and safety should be further investigated.

Development of CAR-M1 (M1: classically activated macrophages) is an emerging therapeutic strategy [88], and several studies of engineered CAR-M cells [89–91] showed tumor cell elimination activity in vitro and in vivo. These studies showed that CAR-M induced phagocytosis and induced M2 to M1 polarization through secretion of pro-inflammatory factors and chemokines [90]. CD47 is ubiquitously expressed on the surfaces of multiple hematopoietic and solid tumor cells. However, adverse events due to cytokine secretion by macrophages during immune checkpoint activation [92, 93] and CAR-T [94, 95] treatment was common. Technological improvements for preparation and production of CAR-M are needed to offer scalable and reproducible manufacturing processes.

### Small molecules, peptides, and microRNA

RRx-001 [96] is an anticancer agent designed to induce M2 to M1 polarization and to promote recovery of phagocytic activity of macrophages toward tumor cells. The anti-phagocytic inhibitory signal was removed or reduced through downregulation of both CD47 and SIRPα gene expression on tumor cells and macrophages, respectively. Elimination of tumor cells was shown in in vitro and in vivo. Phase III clinical trials (NCT03699956, NCT02489903) against small cell lung cancer [97, 98] are ongoing.

D4–2 [99], a macrocyclic peptide targeted to mouse SIRPα was designed to inhibit the interaction between CD47 and SIRPα and promote macrophage-mediated phagocytosis of tumor cells when combined with rituximab. PKHB1 [100], a TSP-1-derived CD47 agonist peptide, induced cell death (CRT exposure and DAMP release) in chronic lymphocytic leukemia cells.

MicroRNAs (miRNAs), which are 20–22 nucleotides in length, play important roles in cancer pathogenesis and progression since they can repress the target gene at the translational level by directly binding to the 3’untranslated regions (3’UTRs) [101].

Overexpression miR-378a [102] in mice peritoneal macrophages downregulates SIRPα mRNA expression. Phagocytosis of Ishikawa cells by macrophages-miR-378a and macrophages was carried out in vitro. Phagocytic index in macrophages-miR-378a group is 3 times than that in macrophages group.

Zhao [103] reported that miR-200a inhibited the expression of CD47 by directly targeting the 3’UTR of the CD47 mRNA. MicroRNA 200a suppressed nasopharyngeal carcinoma (NPC) cell proliferation, migration, and invasion, and promoted phagocytosis of NPC cells by macrophages through down-regulation of CD47 expression on NPC cells.

MicroRNA 708 [104] was directly targeted CD47 and resulted in downregulation of CD47 on T cell acute lymphoblastic leukemia cell line. MicroRNA 708 expression in the T-ALL cell line was sufficient to promote phagocytosis by macrophages in vitro*,* and inhibited tumor engraftment in vivo.

## Conclusions and future perspectives

Following the clinical success of therapeutic antibodies targeting T cell checkpoint molecules, combination therapies using checkpoint inhibitors with other agents have been a major theme of clinical oncology studies. Specifically, inhibitors of the CD47/SIRPα pathway have emerged as promising therapeutic candidates. Overexpression on tumor cells makes CD47 an ideal target for cancer therapy. Antibodies targeting CD47 showed promising results against MDS and AML [105–107]. However, side effects such as anemia, hyperbilirubinemia, thrombocytopenia, and lymphopenia induced by CD47-targeting molecules are of specific concern and need to be addressed with development of new therapeutic agents.

In addition, the therapeutic effects of agents targeting the CD47/SIRPα axis on solid tumors are limited. Additional therapeutic strategies, including combination therapy and bi-specific antibodies, may be promising. Combination therapy with opsonizing antibodies, immune checkpoint inhibitors, chemotherapeutic agents to activate FcR on macrophages, and T cell sensitizers that induce immunogenic cell death to stimulate a more potent immunological effect all have potential. Agents targeted to the CD47-SIRPα axis should not only block the CD47/SIRPα interaction, but also activate signaling on macrophages (FcγR, CRT expression). Other agents including small molecules, mRNA, and CAR-T/M that block the CD47/SIRPα interaction are also in development. Many promising strategies targeting the CD47-SIRPα axis are in development and offer a great deal of hope to patients with cancer.

## Data Availability

Not applicable.

## Abbreviations

ADA: Anti-drug antibody
ADCC: Antibody Dependent Cellular Cytotoxicity
ADCP: Antibody Dependent Cellular Phagocytosis
ADCs: Antibody Drug Conjugates
AML: Acute Myeloid Leukemia
PTPase: Protein Tyrosine Phosphatases
BiAb: Bispecific Antibody
CAR: Chimeric Antigen Receptor
CR: Complete response Rate
CRT: Calreticulin
CTLA-4: Cytotoxic T Lymphocyte-associated Antigen-4
DLBCL: Diffuse Large B-Cell Lymphoma
DLT: Dose-Limited Toxicity
DAMP: Damage Associated Molecular Patterns
GEJ cancer: Gastroesophageal junction cancer
ICIs: Immune Checkpoint Inhibitors
ITIMs: Immunoreceptor Tyrosine-based Inhibition Motifs
MDS: Myelodysplastic Syndrome
MSLN: Mesothelin
NHL: non-Hodgkin’s Lymphoma
ORR: Overall Response Rate
PD-1: Programmed cell Death1
PDCs: Probody Drug Conjugates
PD-L1: Programmed cell Death Ligand 1
PK: Pharmacokinetics
SH2: Src Homology 2
SIRPα: Signal regulatory protein alpha
TME: Tumor Micro-Environment
TRAE: Treatment-related adverse event
VEGF: Vascular endothelial growth factor
